# Re-Epithelialization of Neuropathic Diabetic Foot Wounds with the Use of Cryopreserved Allografts of Human Epidermal Keratinocyte Cultures (Epifast)

**DOI:** 10.3390/jcm11247348

**Published:** 2022-12-10

**Authors:** Fermin R. Martinez-De Jesús, Robert Frykberg, Elízabeth Zambrano-Loaiza, Edward B. Jude

**Affiliations:** 1 The Diabetic Foot Latinamerican Society Research Group, The Diabetic Foot Salvage and Prevention Center Saint Elian, Veracruz 91900, Mexico; 2 Department of Surgery, Midwestern University, Glendale, AZ 85308, USA; 3 Tameside and Glossop Integrated Care NHS Foundation Trust, Ashton-under-Lyne OL6 9RW, UK; 4 The University of Manchester, Manchester M13 9PL, UK; 5 Manchester Metropolitan University, Manchester M15 6BH, UK

**Keywords:** diabetic foot, living skin equivalents, classification, wound healing, keratinocytes

## Abstract

The application of tissue-engineering technology to wound healing has become an option for the treatment of diabetic foot ulcers (DFU). A comparative, prospective study was conducted to assess the efficacy of a cryopreserved allograft of human epidermal keratinocytes (Epifast) to enhance wound healing in granulating DFU. Eighty patients were assigned to receive Epifast (n = 40) or Standard Care (SC) treatment (n = 40). The Epifast group displayed a shorter duration of the epithelialization phase (3.5 ± 4 vs. 6.4 ± 3.6 weeks, *p* < 0.05) and upon the entire wound healing process than the SC group (10 ± 5.7 vs. 14.5 ± 8.9 weeks, *p* < 0.05), reaching wound closure at 16 and 30 weeks, respectively. The Kaplan–Meier analysis revealed that Epifast group patients were 50% more likely than the SC to heal wounds faster (Cox-hazards ratio of 0.5, 95% CI = 0.3–0.8, *p* < 0.0001; Likelihood Ratio of 7.8. *p* < 0.05). Patients in the control group displayed a slower healing as the Saint Elian (SEWSS) severity grade increased (group differences of 0.6, 3.8, and 4.3 weeks for grades I, II, and III, respectively). DFW treated with Epifast displayed a shorter time to complete re-epithelialization than wounds treated with standard care.

## 1. Introduction

Foot ulcerations are among the most complex and heterogeneous complications in diabetic patients. The application of tissue-engineering technology to wound healing has become an option for the treatment of chronic wounds [1,2,3,4,5,6]. Skin equivalents are constructed from cultured keratinocytes that form an epidermal layer without dermal components. Epifast promotes cellular migration and produces growth factors that stimulate cell proliferation from ulcerated skin to achieve re-epithelialization [7].

Many factors play a role in the wound healing process, such as foot pressure, ischaemia, growth factors and cytokines and infection [ref]. Based on our experience with a previously published grading system [8], one important factor for wound progress is the wound healing phase. Clinical trials usually assess the efficacy of living skin equivalents in terms of percent of wound healing and frequency of amputation. We propose that their wound healing efficacy is useful during the granulation phase. In this study, we hypothesize that Epifast enhances re-epithelialization and reduces wound healing time in patients with diabetic foot wounds. Thus, we assessed the efficacy of an allograft of human epidermal keratinocytes (Epifast) to shorten the time to achieve 100% of wound closure and the epithelialization healing time. (A period started when granulation tissue is completed and re-epithelialization from the wound border begins).

## 2. Materials and Methods

### 2.1. Patients

A randomised, comparative, prospective study was undertaken to evaluate the effect of Epifast in DFUs. Consecutive type 2 diabetes patients from the foot clinic at our center with diabetic foot wounds were screened for eligibility for the study but included by random allocation when granulation tissue was in progress. Patients with type 2 diabetes who were over 18 years of age and had wounds at or distal to the malleoli with different degrees of neuropathy, anatomic, and tissue affection were considered for inclusion. An ulcer was defined as a full skin thickness wound below the ankle in a diabetic patient, irrespective of their etiology or duration. Only patients with non-infected, granulating wounds at or distal to the malleoli with loss of protective sensation and at least one pedal pulse detected by Doppler were included. Wound size was evaluated by measuring the maximum length and the maximum width. (When >1 ulcer was present only the largest ulcer was included).

Exclusion criteria included the following: (a) arterial disease (diagnosed by the absence of both foot pulses on the affected extremity, an ankle/brachial index below 0.5, or toe pressure < 30 mm Hg and a toe/brachial index < 0.30); (b) osteomyelitis and/or total gangrene of the foot or forefoot; (c) severe cardiovascular and renal failure; (d) severe neurological problems; (e) and other situations that would make the patient a poor candidate for the study (e.g., confined to a bed or lacking family assistance). All patients provided written informed consent. The study was reviewed and approved by the Human Subjects Ethics Committee.

### 2.2. Protocol

Once the granulating wound healing phase was determined by clinical inspection; all eligible subjects were randomly assigned to receive either Epifast (Group 1) or continued under standard management (control Group 2). Patients were blinded with respect to treatment and were informed only that a high-tech dressing could be randomly selected for their treatment. Both groups received comprehensive care and evaluation. All patients were treated according to an outpatient ambulatory model that included appropriate surgical debridement, aggressive parenteral/intramuscular broad spectrum antimicrobial administration, appropriate off loading, and strict glycemic control. Patients in both treatment groups were seen every third day or once a week. All patients were instructed to use a wheelchair or crutches to reduce load bearing on the affected foot and to rest as much as possible, although most patients have difficulty complying with these instructions. Compliance was assessed by directly questioning the patient and their caretaker, as well as by inspecting the dressings.

Patients randomized to the study group received the keratinocyte skin culture application (Epifast^®^; Bioskinco, Mexico City, México) [7]. Epifast is free from HIV-1, HIV-2, HBV, CMV, and toxic substances. The Epifast allografts were placed over a sterile tissue mesh between two protective plastic nets (7.00 × 8.00 cm, total surface of 56 cm^2^). The allografts were then applied as a dressing over the wounds and changed every 7 days for one month. Patients were advised about malodor not related to infection.

Subjects under standard care treatment received hydrofiber, alginate, or Vaseline dressings. Alginate or hydrofiber dressings were used when the wound was exudative. Vaseline dressings were used during the non-exudative granulation phase until complete wound healing occurred.

### 2.3. Primary Objective and Measurements

The primary endpoint was the duration in weeks for the epithelialization phase to achieve 100% closure of the wound, which was measured from the advance of new skin from the baseline wound border until complete healing. Epithelialization was defined as the proliferation of epithelial cells providing cover for the new granulation tissue to restore an intact epidermal barrier. Granulation tissue is pink/red, moist tissue composed of new blood vessels, connective tissue, fibroblasts, and inflammatory cells that fills an open wound during healing. Epifast was applied while the granulation phase was in progress. Changes in the surrounding skin and granulation tissue were assessed by direct clinical observation. The presence of healthy tissue surrounding the ulcers was considered a clinical sign that tissue toxicity was absent or minimal. Outcomes were assessed over a mean follow-up period of 52 weeks (365 days). The follow-up period was part of the normal treatment duration according to healing success or failure and a secondary follow-up with a minimum of 6 months for delayed wound healing. Once ulcer healing was achieved, patients were released from the study.

Baseline demographic measurements were performed at the first visit. The diagnosis of diabetes was made prior to enrollment. Doppler studies were performed, and toe or ankle/brachial indices were calculated. Pulse palpation, ABI (Hand-held Doppler– Huntleigh, Getinge AB; 8 MHz Doppler probe) and TBI were performed sequentially to assess the vascular status in every patient included in the study. Toe pressure was measured using a 2.5 cm wide × 12 cm long digital cuff on the proximal aspect of the hallux to calculate the TBI. A PPG unit Hadeco Smartdop 30EX was used when no toe artery was found using a Doppler and ischemia severity was graded as normal (0), mild (1), moderate (2) and severe (3). The degree of neuropathy was assessed using a 128-Hz vibration from a tuning fork at the hallux or on the basis of a decrease in or absence of Semmes–Weinstein (5.7/10-g) monofilament sensation at 2 of 3 points (first toe and fifth and first metatarsal head). Neuropathy for severity score purposes was sub-grouped as (0) none, (1) mild (diminished protective sensation to vibration or monofilament), (2) moderate (absence of sensation to vibration or monofilament), and (3) severe when diabetic neuro-osteoarthropathy (DNOA) was found. Infection was assessed and scored according to the Infectious Disease Society of América as part of the Saint Elian System [8]. Infection was excluded and scored as (0) without signs or symptoms. Mild infection affected skin superficially and was scored as 1 and diagnosed as erythema between 0.5 mm and 2 cm, induration, tenderness, warmth, and purulent discharge. Moderate infection (scored as 2) was identified by erythema more than 2 cm, muscle, tendon, bone, or joint infection. Osteomyelitis was diagnosed by a positive bone-to-probe test, by X-ray film, or biopsy. Severe infection (scored as 3) was identified by systemic inflammatory response or severe metabolic disturbances (hyperglycemia or hypoglycemia) that required patient hospitalization or was difficult to control. Neuropathy, infection and vascular assessments and wound characteristics were included as part of the Saint Elian Wound Score System for the Diabetic Foot [8,9]. According to this score, the 10 wound variable categories measured at patient presentation were as follows: (1) primary location, (2) topographic aspects, (3) number of affected zones, (4) ischemia, (5) infection, (6) edema, (7) neuropathy, (8) depth, (9) area, and (10) wound healing phase. All categories were subcategorized with an ascending score from mild (1) to severe (3 points).

The maximum score was 30 points. A score of 10 points or fewer was graded as I (mild, likely successful wound healing). A moderate score of 11–20 points was graded as II (partial foot threatening; outcome related to “state of the art” therapies used and associated with a good patient response). A score of 21–30 points was graded as III (limb- and life-threatening; outcome unrelated to “state of the art” therapies due to a poor patient response).

Consecutive measurements were recorded on different dates with the following variables in the left column: date, therapy, the ten wound variable categories, score, cumulative difference, and grade. Data were registered according to the date for each item as many times as needed.

Wound images and wound measurements were recorded in a database for systematic data collection.

### 2.4. Statistical Analysis

Significance was considered at *p* < 0.05. Values for chi-squared with Yate’s correction or Fisher’s exact test with 2 × 2 tables and variance ratios for natural and treatment analysis of variance were calculated. Kaplan–Meier probability, log-rank test, and Cox Hazard Ratio analyses were performed for wound healing time and were measured against the study groups. Simple regression was performed for variables such as wound size and time assuming a dependent relationship of wound size during the time period of examination. The p value for slopes differences was determined by Student’s *t*-test (two-tailed). The sample size was required to have 80% power to detect differences for a one-sided test hypothesis: H_o_: P_1_ = P_2_ versus H_a_: P_1_ < P_2_ or P_1_ > P_2_. A 5% α (0.05) significance level, an 80% (0.80) power test (1-β), a 90% (0.90) success proportion in arm 1 (P_1_), an 80% (0.80) success proportion in arm 2 (P_2_), and a 1:1 group ratio were set for calculations. A total sample size for arm 1 and 2 of 68 patients was required, with 34 patients in each group. Forty patients per group were included, assuming a 20% drop-out rate. Patients assessed for eligibility were submitted to block randomization with an allocation ratio of 1:1. Two members of staff scored the Saint Elian System and assessed the epithelialization phase and healing progress independently. Kappa agreement index was performed using 2 × 2 tables to detect differences between the two observers. A kappa value (K ¼ Po−Pe/1−Pe; Po ¼ observed agreement, Pe ¼ expected agreement by random) between 0.61 and 1.0 was representative of substantial to excellent agreement.

## 3. Results

**Figure 1:**
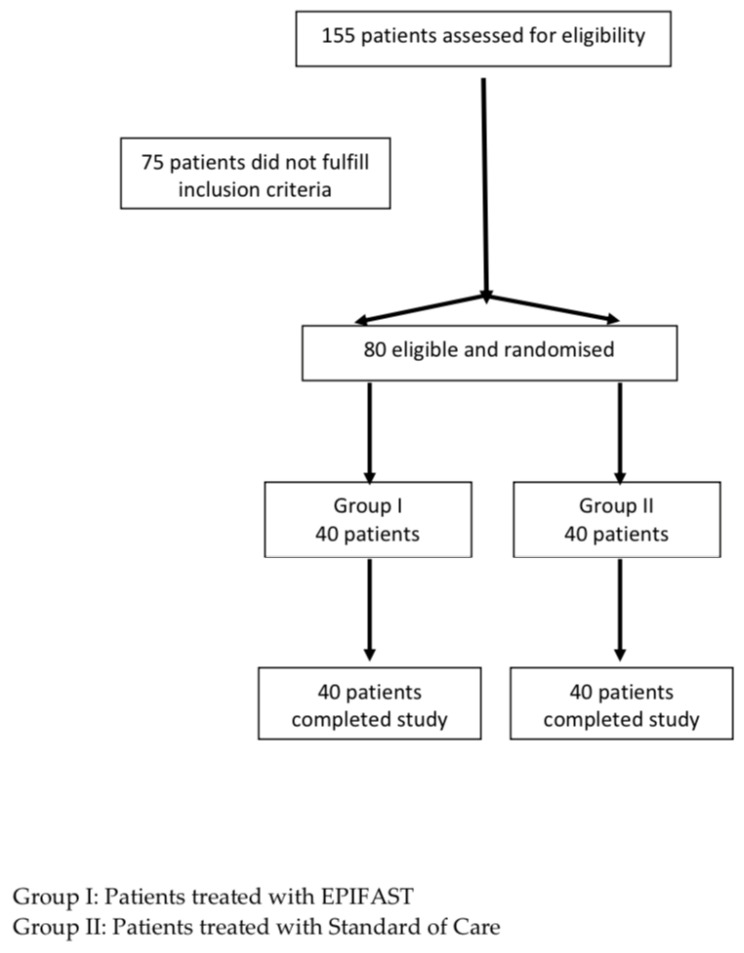
Flowchart showing patient randomisation.

Of 155 patients initially assessed, 75 were excluded because of the patient’s decision, the refusal to complete the protocol or the failure to achieve the granulation healing phase (Figure 1). A total of 80 patients were included and were randomized to receive either Epifast (n = 40, Group 1) or standard care (n = 40, Group 2).

There were no differences in demographics and clinical characteristics between the groups (Table 1). The 10 categories and subcategories for wound severity (Saint Elian classification) displayed similar proportions among the groups. The wound duration (lapse from the initial wound to patient presentation) was 7.2 (range 3–15) weeks. The treatment duration was 9.0 weeks (range 1–30). Healing times according to the initial assessment of the San Elian severity grades were 6.0 (range 1–35), 10.4 (range 1–122) and 26.8 (range 1–64) weeks for grades I, II and III, respectively (*p* < 0.05).

Ulcer size in cm^2^ was similar between the two groups (11.1 range (2.5–32.5) vs. 12.2 (range 3–28) cm^2^, *p* > 0.05, respectively, Table 1). The Saint Elian severity grades and score sums initially performed displayed no differences in distribution between groups for grades I through III.

Although total wound healing was 10% better for Group I (95 vs. 85%, respectively; Fisher’s exact test), this difference (*p* = 0.09) was not significant (Table 2). Three patients in Group 1 and six patients in Group 2 did not heal. Patients included in Group 1 (Table 2) healed faster than those in Group 2 (10 ± 5.7 vs. 14.5 ± 8.9 weeks, *p* < 0.05; respectively), with a shortened duration of epithelialization phase (3.5 ± 4 vs. 6.4 ± 3.6 weeks, *p* <0.05).

**Figure 2:**
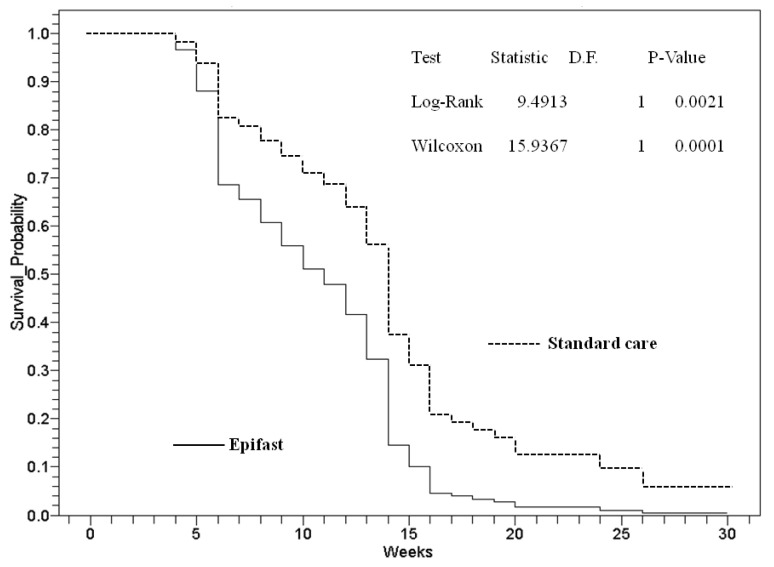
Kaplan–Meier survival probability analysis of wound healing failure by weeks for study groups with a mean follow-up period of 365 days. Survivors were non-healing patients.

The Kaplan–Meier survival probability analysis revealed significant differences in healing times between Group 1 and Group 2 (Figure 2).

Cox proportional hazards showed that Group 1 had a 50% faster healing time than the standard care group (Hazard ratio 0.5, 95% CI = 0.3–0.8; *p* < 0.01), with a likelihood ratio of 7.8 for delayed healing in Group 2 (*p* < 0.05). The Epifast group healed faster at 16 weeks versus the SC group that reached 100% wound closure by 30 weeks (y = −1.6429x +14.571; r = 0.94464 and y = −2.5x +12.5; r = 0.89928, respectively).

At 52 weeks of follow-up, one patient from Group 1 developed re-ulceration at a different location. No cases of re-ulceration occurred in Group 2. Excessive granulation tissue was observed in eight patients in Group 1 and 2 patients in Group 2 (*p* < 0.05). The excessive granulation tissue was removed surgically as many times as necessary (mean, 4.2 ± 3.2 weeks). After complete re-epithelialization, no further excessive granulating activity was observed during the follow-up period.

In Group 1, one patient had major amputation secondary to severe acute renal failure (ARF), remaining chronically wounded after the end of the study follow up (two patients) and reinfection (one patient). Patients in Group 2 failed to heal due to reinfection (four patients), and death secondary to ARF (one patient), and major amputation (one patient).

## 4. Discussion

This is the first study to show improved wound healing with application of Epifast to the wound in patients with non-healing DFUs compared to standard care. Overall patients in the former group had improved wound epithelialization and significant faster time to healing.

Our study reports an average healing time of 67 days for Group 1 vs. 97 days for the control. In Group 2, the completeness of wound closure (95% for Epifast vs. 85% for standard care) was similar but slightly superior to a previous study of Dermagraft which reported a median percent wound closure of 91% compared to 78% for the control group [6].

In the present study, we showed that patients treated with Epifast healed faster than the standard care group due to shortened re-epithelialization time of diabetic foot ulcers. The healing time was reduced compared to the control group. The similar proportions achieved for the higher wound healing success rates in both study groups indicates that patients included in our study were randomly assigned during the advanced granulation phase, that was not included within the results and discussion of the Dermagraft study. During this phase, infection, ischemia, or other aggravating factors were not present due to good treatment response achieved prior to randomization with strong progress towards wound healing.

Patients included in the present study by random assignment were a selected sample with a high probability of healing and then randomized to active or control groups. Even those patients with severity grades of III showed good progress toward healing at the time of randomization. As we reported previously, this response is achieved in only 30% of grade III patients [8].

We also showed that Epifast enhanced epithelialization to varying extents according to wound severity. Differentiating wound severity (mild, moderate and severe) was important for analyzing the time to healing and was used as a confounder to control bias in group comparisons. Epifast shortened the wound healing time compared with the control group as wound severity increased. The severity of wounds was graded using the Saint Elian Scoring System [8,9] avoiding limitations and erroneous interpretations when documenting diabetic foot ulcers characteristics [10,11]. Conversely, the SEWSS internal and external validation reports an inter-observer analysis with high concordance between two observers [8,9]. In the present study, the observers showed a high Kappa agreement index of 0.88 and 0.92 to score categorization and grade of the Saint Elian system and for the assessment of healing progress during re-epithelialization, respectively.

Different biological treatments have been investigated to facilitate wound healing in patients with diabetic foot ulcers, such as Leucopatch [12], stem cell therapy [13,14], and Dermagraft [6] with varying results. Recent advances in stem cell therapy using mesenchymal stem cells and pluripotent stem cells is promising [15,16], but has not been translated into better wound healing and diabetic foot outcomes [17]. The differences in the biological characteristics of human skin equivalents compared to natural skin are due to the autologous nature of human skin equivalents, as well as their structure and composition of a mixture of biological and non-biological substances that are categorized as epidermal, dermal, and full-thickness skin substitutes. Epifast, which belongs to the first category, is a cryopreserved allograft of neonatal human epidermal foreskin cultured keratinocytes that constitute an epidermal layer without dermal components [7]. The use of keratinocytes stimulate migration of native keratinocytes from the wound edge [18]. A cell-based therapy is a highly promising approach for DFU treatment for ischemic DFU including mesenchymal stem cells that can be effective to provide an adjuvant therapy for limb salvage. The superiority of some specific cell types for DFU treatment is controversial. Undoubtedly, cell therapy is a potent tool for the treatment of DFU. However, further high-quality clinical research to determine the most effective cell type for DFU treatment must be conducted.

### 4.1. Side Effects

Our results indicate an incidence of benign hyper-granulation tissue. This condition is likely a side effect related to growth factor activity that enhances the formation of granulation tissue. Growth factors and extracellular matrix proteins are present in the frozen cultured sheets of human epidermal keratinocytes used during wound healing [18]. Although cancer risk has been reported in patients treated with recombinant human platelet-derived growth factor [19], hyper-granulation tissue in the present study was completely benign.

### 4.2. Study limitations

This trial was a single blind study that compared standard care instead of placebo, in contrast to a double-blind trial design. Although sample size calculations were made, the lack of significant results for rates of total wound healing (*p* equal to 0.09) can be explained by type II error (the null hypothesis is not rejected when it is in fact false) and must be addressed by increasing the sample size in future studies. For Kaplan–Meier analysis, a minimum sample size of 30 patients is required; therefore, this test assumption was satisfied. However, a multicenter study with a bigger sample size could help to strength these preliminary results.

## 5. Conclusions

**Figure 3:**
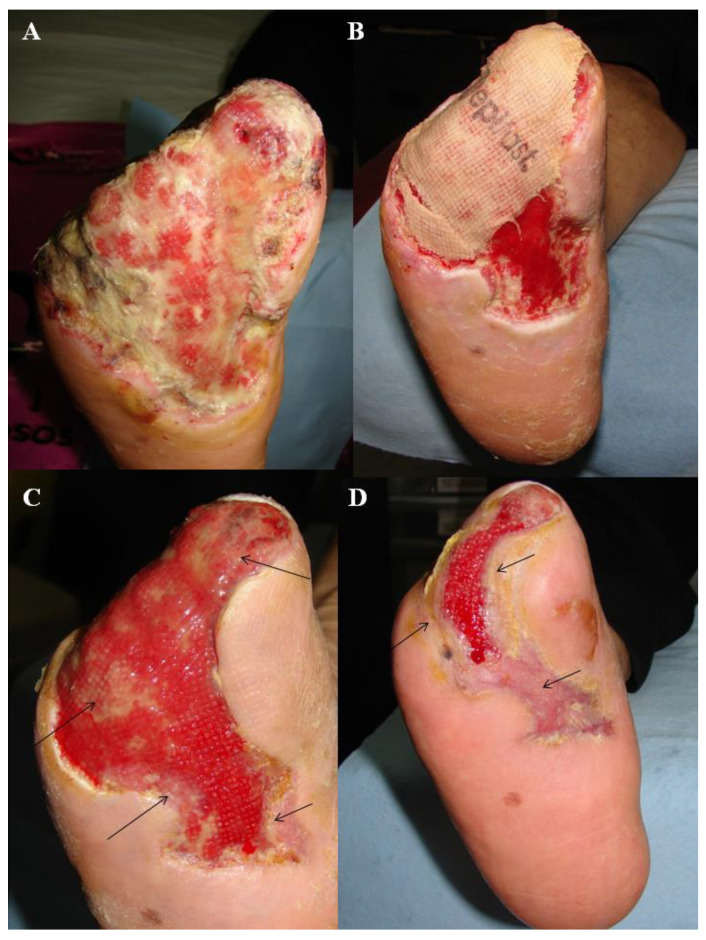
(**A**) 100 cm^2^ complex, difficult to heal diabetic foot wound in the granulation phase after random assignment to the Epifast group. (**B**) A granulating wound covered by Epifast after 3 weeks, enhanced by growth factors present in the Epifast. (**C**) Arrows indicate newly formed skin 8 weeks after treatment. (**D**) Spread of epithelialization after 16 weeks. Total epithelialization was achieved 2 weeks later.

In conclusion, we have demonstrated significantly faster healing in patients with diabetic foot ulcers that were treated with human epidermal keratinocytes (Epifast) than standard care due to better re-epithelialization of the ulcers (Figure 3). Our results might be extended to those patients who achieve a granulation phase and are prone to complete re-epithelialization and therefore could facilitate faster wound healing.

## Figures and Tables

**Table 1:** Baseline demographic and clinical characteristics.

Characteristic	Group 1 Epifast n = 40 (%)	Group 2 Standard Care n =40 (%)	*p* Value
Age yr means ± SD +	65 ± 11.8	63.1 ± 11.3	0.42
Sex *			0.20
Male	22 (52.5)	19 (47.5)	
Female	18 (45)	21 (52.5)	
Diabetes duration in years means ± SD +	18.8 ± 11.3	18.7 ± 9.7	0.70
HbA1c means ± SD, % [mmol/mol] **	8.9 ± 1.9 [74]	8.2 ± 2.3 [66]	0.64
Obesity	26 (65)	30 (75)	0.54
Smoking *	6 (28)	9 (30.3)	0.33
Palpable peripheral pulses *	40 (100)	40 (100)	
Wound history in weeks means ± SD +	4.7 ± 6.1	6 ± 7.6	0.49
Saint Elian Score means ± SD+	19 ± 1.6	16 ± 2.0	0.81
Saint Elian Severity Grades *			0.60
I (good prognosis for wound healing)	6 (15)	5 (12.5)	
II (partially foot-threatening)	30 (75)	33 (82.5)	
III (limb- and life-threatening)	4 (10)	2 (5)	

+ Kruskal–Wallis. * Chi-squared ** IFCC standardization system (NGSP = [0.09148 × IFCC] + 2.152).

**Table 2:** Average duration of epithelialization, wound healing and severity grades for study groups.

Outcomes	Group 1 Epifast n = 40 (%)	Group 2 Standard Care n = 40 (%)	*p* Value
Complete wound healing *	38 (95)	34 (85)	0.09
Time to healing [weeks mean ± SD] **	10 ± 5.7	14.5 ± 8.9	0.003
Epithelialization time [wks mean ± SD] **	3.5±4	6.4 ± 3.6	0.001
Wound healing by severity grades (SEC) [wks mean ± SD] ***			
Grade I	1.7 ± 0.4	2.3 ± 3.0	0.003
Grade II	2.3 ± 2.0	6.1 ± 3.0	
Grade III	12.2 ± 3.3	16.5 ± 3.4	

(SEC) = Saint Elian Classification * Fisher’s exact test ** Kruskal–Wallis *** ANOVA.

## Data Availability

Data supporting reported results can be found within the archives of the clinical charts in our center.
